# Blood pressure trajectories during pregnancy and preterm delivery: A prospective cohort study in China

**DOI:** 10.1111/jch.14494

**Published:** 2022-06-01

**Authors:** Fanfan Chan, Songying Shen, Peiyuan Huang, Jianrong He, Xueling Wei, Jinhua Lu, Lifang Zhang, Xiaoyan Xia, Huimin Xia, Kar Keung Cheng, Shakila Thangaratinam, Ben Willem Mol, Xiu Qiu

**Affiliations:** ^1^ Division of Birth Cohort Study Guangzhou Women and Children's Medical Center Guangzhou Medical University Guangzhou China; ^2^ Provincial Key Clinical Specialty of Woman and Child Health Guangdong China; ^3^ Provincial Clinical Research Center for Child Health Guangdong China; ^4^ Institute of Applied Health Research College of Medical and Dental Sciences University of Birmingham Birmingham UK; ^5^ WHO Collaborating Centre for Women's Health Institute of Metabolism and Systems Research University of Birmingham Birmingham UK; ^6^ Department of Obstetrics and Gynecology School of Medicine Monash University Melbourne Australia

**Keywords:** blood pressure, iatrogenic preterm delivery, pregnancy, preterm delivery, spontaneous preterm delivery

## Abstract

Women's blood pressure (BP) changes throughout pregnancy. The effect of BP trajectories on preterm delivery is not clear. The authors aim to evaluate the association between maternal BP trajectories during pregnancy and preterm delivery. The authors studied pregnant women included in the Born in Guangzhou Cohort Study in China between February 2012 and June 2016. Maternal BP was measured at antenatal visits between 13 and 40 gestational weeks, and gestational age of delivery data was collected. The authors used linear mixed models to capture the BP trajectories of women with term, and spontaneous and iatrogenic preterm delivery. BP trajectories of women with various gestational lengths (34, 35, 36, 37, 38, 39, 40 weeks) were compared. Of the 17 426 women included in the analysis, 618 (3.55%) had spontaneous preterm delivery; 158 (.91%) had iatrogenic preterm delivery; and 16 650 (95.55%) women delivered at term. The BP trajectories were all J‐shaped curves for different delivery types. Women with iatrogenic preterm delivery had the highest mean BP from 13 weeks till delivery, followed by those with spontaneous preterm delivery and term delivery (*p *< .001). Trajectory analysis stratified by maternal parity showed similar results for nulliparous and multiparous women. Excluding women with pre‐eclampsia and gestational hypertension (GH) significantly attenuated the aforementioned association. Also, women with shorter gestational length tend to have higher BP trajectories during pregnancy. In conclusion, Women with spontaneous preterm delivery have a higher BP from 13 weeks till delivery than women with term delivery, while women with iatrogenic preterm delivery have the highest BP.

## 1 INTRODUCTION

Preterm delivery, defined as delivery before 37 completed weeks of gestation, is the leading cause of perinatal and neonatal morbidity and mortality.^1^ Preterm delivery affected about 10.6% live births based on the worldwide estimate in 2014.^2^ It contributes to various short‐ and long‐term adverse offspring outcomes including neonatal death, respiratory distress, cerebral palsy, feeding difficulties, visual disorders and needs for prolonged and repeated hospitalization,^3^, ^4^, ^5^ exposing family and the health system to tremendous economic burden.^6^, ^7^ So far, approximately 70% in preterm delivery are spontaneous preterm delivery and the remains are iatrogenic preterm delivery.^1^

It is suggested that placenta dysfunction may partially be responsible for preterm delivery.^8^, ^9^, ^10^, ^11^ Improper remodeling of the uterine spiral arteries may lead to worse placenta perfusion and subsequent maternal vascular endothelium dysfunction, which may result in further preterm delivery.^8^, ^9^ In addition, Kim and colleagues^12^, ^13^ indicated that failure of physiologic transformation of spiral arteries is more frequent for preterm delivery than term delivery. All things considered, nonoptimal physiologic transformation of spiral arteries and subsequent worse placenta perfusion may result in complementary increased blood pressure (BP) to support fast‐growing fetus. Thus, it is reasonable to assume that maternal BP during pregnancy is related to preterm delivery.

Women experience considerable changes of BP during pregnancy, with BP decreasing until about 18 weeks of gestation and then increasing until delivery.^14^ However, the understanding of the relationship between maternal BP change during pregnancy and preterm delivery is limited. The Avon Longitudinal Study of Parents and Children showed that smaller decrease in BP before 18 weeks and greater increase between 18 and 36 weeks were associated with shorter gestation.^15^ Yet it was unable to distinguish between spontaneous and iatrogenic preterm delivery. Zhang and colleagues^16^ found that an excessive rise in BP from early pregnancy (12∼19 gestational week) to midthird trimester (30∼34 gestational week) was related to spontaneous preterm delivery in a dose‐response manner. Nevertheless, this study focused on women with spontaneous preterm delivery without detailed investigation of the association between all preterm delivery and BP change. All in all, the few relevant studies were unable to explore the relationship between BP change and all preterm delivery subtypes. A placenta histology study indicated that both spontaneous and iatrogenic preterm delivery have maternal vascular malperfusion lesions but are different in terms of severity,^17^ which suggests that these two subtypes may be similar to some extent. Therefore, it may not be appropriate to simply exclude either spontaneous or iatrogenic preterm delivery.

We aimed to determine whether trajectories of BP during pregnancy is related to spontaneous and iatrogenic preterm delivery in a Chinese population.

## 2 METHODS

The present study was part of the Born in Guangzhou Cohort Study, a prospective cohort study performed in Guangzhou Women and Children's Medical Center, China. Its recruitment procedure and study protocol have been described elsewhere.^18^ Briefly, during the first antenatal visit in Guangzhou Women and Children's Medical Center, women were invited to join Born in Guangzhou Cohort Study if they were less than 20 weeks pregnant and intended to deliver at Guangzhou Women and Children's Medical Center. At enrollment and subsequent antenatal care visits, participants were asked to complete questionnaires on participants’ characteristics.

Women were eligible for this study if they had a singleton pregnancy. Women were excluded if they had pre‐existing hypertension, pre‐existing diabetes, abortion or stillbirths. Women with missing information on gestational age at delivery and preterm category were excluded. Also, women were excluded if they had less than three BP measurements from gestational week of 13–40. The study protocol was approved by the Institutional Ethics Committee of the Guangzhou Women and Children's Medical Center. Written informed consent was obtained from all participants before participating in Born in Guangzhou Cohort Study.

Maternal BP was measured at each antenatal visit (between 13 and 40 weeks of gestation) using an automatic BP monitor (OMRON HBP‐9020, Kyoto, Japan) and was entered directly into participants’ antenatal care records. All participants relaxed for at least 5 min and were seated in an upright position. A cuff was positioned at the level of the heart on the right upper arm. BP measurements were considered outliers if they were over five standard deviations (SD) away from the mean level at each gestational week.

Gestational age, obtained from medical records, was based on women's last menstrual period and confirmed by ultrasound in the first/early second trimester. Preterm delivery, defined as delivery before 37 weeks of gestation, was subdivided into spontaneous and iatrogenic preterm delivery. Spontaneous preterm delivery was defined as deliveries following spontaneous preterm labor and/or preterm premature rupture of the membranes and iatrogenic preterm delivery as deliveries following induced labor or prelabor cesarean delivery, performed on account of maternal and/or fetal reasons, for instance, pre‐eclampsia (PE) and fetal growth restriction.^1^

Maternal characteristics like age (years), height (cm), maternal income (≤1500/1501–4500/4501–9000/≥9001 yuan), educational level (high school or below/vocational/technical college/undergraduate/postgraduate), parity (primiparous/multiparous),and presence of factors such as maternal depression and anxiety during pregnancy, smoking during pregnancy, passive smoking, family history of hypertension, history of preterm birth, vaginal bleeding, and folic acid supplementation during pregnancy were obtained from the questionnaire at first antenatal care visit. Data on gestational diabetes (GDM), PE, gestational hypertension (GH), cesarean section and infant sex were obtained from medical records. Prepregnancy body mass index (BMI) was calculated as pre‐pregnancy weight divided by height squared.

First, we presented the participant characteristics in mean ± SD and counts (percentage) for continuous and categorical variables, respectively, by delivery types (term delivery, iatrogenic and spontaneous preterm delivery). Analysis of variance (ANOVA) and chi‐square were performed to test differences for categorical and continuous variables, respectively. Second, based on Akaike information criterion (AIC), fractional polynomial were conducted to ascertain the best fitting median trajectory.^19^ The best‐fitting fractional polynomials were ${\left(\frac{\mathrm{gestational}\;\mathrm{age}\;\mathrm{at}\;\mathrm{measurement}}{10}\right)}^{-2}$ and ${\left(\frac{\mathrm{gestational}\;\mathrm{age}\;\mathrm{at}\;\mathrm{measurement}}{10}\right)}^{3}$ for systolic BP, ${\left(\frac{\mathrm{gestational}\;\mathrm{age}\;\mathrm{at}\;\mathrm{measurement}}{10}\right)}^{.5}$ and ${\left(\frac{\mathrm{gestational}\;\mathrm{age}\;\mathrm{at}\;\mathrm{measurement}}{10}\right)}^{3}$ for diastolic BP. The results of fractional polynomial regressions were further modeled as fixed effects in mixed‐effects models. These models could take into account the correlation among repeated BP measurements within individual. In addition, intercept and gestational age at BP measurement were included as random effects. Moreover, to take into account the effects of covariates and potential confounders on the BP trajectories, all mixed‐effects model were adjusted for maternal age, pre‐pregnancy BMI, smoking and passive smoking during pregnancy, parity, maternal income level and education level, infant's sex and history of preterm delivery. In addition, additional interaction terms between delivery type and the aforementioned transformations on gestational age at BP measurements did not show any improvement in the goodness of fit, which were not incorporated into mixed‐effects models. Building on mixed‐effects model, penalized B‐splines were applied to ascertain the average BP trajectories for different delivery types. In addition, to correct for baseline BP at 13 weeks of gestation, BP change from 13 weeks of gestation during pregnancy was calculated as the differences between each week and 13 weeks of gestation, and modeled to generate the trajectories of BP change during pregnancy. Since maternal parity is associated with the risk of preterm delivery in subsequent pregnancies,^20^, ^21^, ^22^ we further did the abovementioned trajectory analysis for nulliparous and multiparous women separately. Third, with PE and GH being the most common risk factors for preterm delivery, we did the trajectory analysis aforementioned after excluding women with PE and GH. Fourth, building on the aforementioned mixed‐effects models, penalized B‐splines were applied to ascertain the average BP trajectories for different gestational lengths (34, 35, 36, 37, 38, 39, and 40 weeks of gestation).

All statistical analyses were performed with the Statistical Analysis System version 9.4 (SAS Institute Inc, Cary NC) and R (version 4.0.5) for Windows. A two‐tailed *p‐*value <.05 was considered statistically significant.

## 3 RESULTS

Between February 2012 and June 2016, we recruited 25 010 women into the Born in Guangzhou Cohort Study, of which we excluded 7584 women (women who withdrew before delivery [*n* = 1156], women with multiple pregnancies [*n* = 506], abortion or stillbirths [*n* = 295], pre‐existing hypertension [*n* = 37], pre‐existing diabetes [*n* = 57], incomplete information on delivery [*n* = 949] and women with <3 BP measurements between 13 and 40 weeks of gestation [*n* = 4584]). There were 17 426 women with living singleton deliveries remained in the final analysis (Supplementary Figure S1).

Of 17 426 deliveries, the rate of overall preterm delivery was 4.45% (776/17426), with the rate of spontaneous and iatrogenic preterm delivery 3.55% (618/17426) and .91% (158/17426), respectively. Women with preterm delivery tended to be older, have higher pre‐pregnancy BMI, be more anxious and depressive during pregnancy, be more likely to expose to smoking, have a higher rate of history of preterm delivery, cesarean section, PE, GH and vaginal bleeding during pregnancy. The number of BP measurements was 9.36 ± 2.21 for term delivery, which was significantly larger than those for spontaneous preterm (6.67 ± 1.90) and iatrogenic preterm (6.66 ± 2.08), respectively (Table 1).

**TABLE 1:** Characteristics of subjects for the analysis of blood pressure during pregnancy and gestational length

Characteristics	Term delivery (n = 16 650)	Spontaneous preterm delivery (n = 618)	Iatrogenic preterm delivery (n = 158)	*p*
Mother				
Age, years	29.87 ± 3.75	30.24 ± 3.75	30.97 ± 3.87	<.01
Height, cm	159.97 ± 4.92	159.21 ± 4.68	159.14 ± 4.56	<.01
Income, %				
≤1500	1464 (8.79)	64 (10.36)	15 (9.49)	.49
1501–4500	4156 (24.96)	152 (24.60)	32 (20.25)	
4501–9000	6668 (40.05)	246 (39.81)	74 (46.84)	
≥9001	3475 (20.87)	120 (19.42)	32 (20.25)	
Missing	887 (5.33)	36 (5.83)	5 (3.16)	
Education, %				
High school or below	1525 (9.16)	66 (10.68)	18 (11.39)	<.01
Vocational/technical college	3960 (23.78)	156 (25.24)	43 (27.22)	
Undergraduate	9029 (54.23)	306 (49.51)	79 (50.00)	
Postgraduate	2136 (12.83)	90 (14.56)	18 (11.39)	
Pre‐pregnancy body mass index, kg/m2, Mean ± SD	20.52 ± 2.73	20.74 ± 2.90	21.06 ± 3.26	<.01
Maternal depression at early pregnancy, %	3134 (18.82)	111 (17.96)	30 (18.99)	<.01
Maternal anxiety at early pregnancy, %	2251 (13.52)	75 (12.14)	24 (15.19)	<.01
Smoking during pregnancy, %	68 (.41)	3 (.49)	3 (1.90)	<.01
Passive smoking during pregnancy, %	4740 (28.47)	157 (25.40)	67 (42.41)	<.01
Multiparous, %	4468 (26.83)	138 (22.33)	43 (27.22)	<.01
Family history of hypertension, %	4397 (26.41)	176 (28.48)	46 (29.11)	<.01
History of preterm delivery, %	258 (1.55)	27 (4.37)	9 (5.70)	<.01
Vaginal bleeding during pregnancy, %	4582 (27.52)	223 (36.08)	55 (34.81)	<.01
Folic acid supplementation, %	14135 (84.89)	524 (84.79)	137 (86.71)	<.01
Cesarean section, %	5417 (32.53)	165 (26.70)	143 (90.51)	<.01
Pre‐eclampsia, %	108 (.65)	4 (.65)	57 (36.08)	<.01
Gestational diabetes, %	2352 (14.13)	115 (18.61)	32 (20.25)	<.01
Gestational hypertension, %	365 (2.19)	15 (2.43)	4 (2.53)	<.01
Number of BP measurements	9.36 ± 2.21	6.67 ± 1.90	6.66 ± 2.08	<.01
Infant				
Birthweight, g, Mean ± SD	3218.54 ± 376.90	2500.45 ± 430.89	2231.04 ± 581.72	<.01
Gestational weeks at delivery, weeks, Mean ± SD	38.87 ± .92	34.98 ± 1.57	34.51 ± 1.79	<.01
Infant sex,%				
Male	8680 (52.13)	380 (61.49)	82 (51.90)	<.01
Female	7970 (47.87)	238 (38.51)	76 (48.10)	

Abbreviations: BMI, body mass index; BP, blood pressure; SD, standard deviation.

**FIGURE 1:**
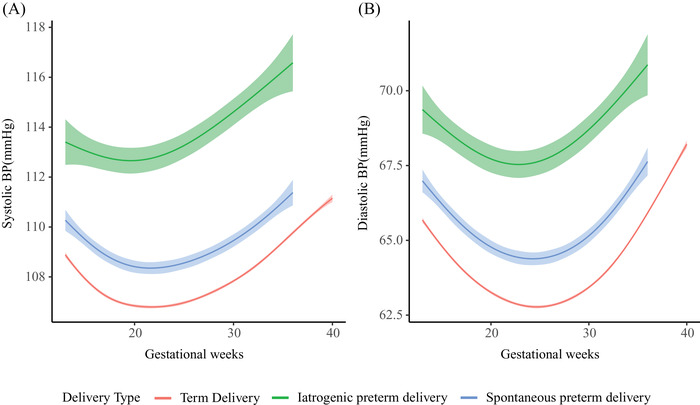
Blood pressure trajectories among women with iatrogenic and spontaneous preterm and term delivery. All models were adjusted for maternal age, pre‐pregnancy BMI, smoking and passive smoking during pregnancy, parity, maternal income level and education level, infant sex and history of preterm delivery. BP, blood pressure

**FIGURE 2:**
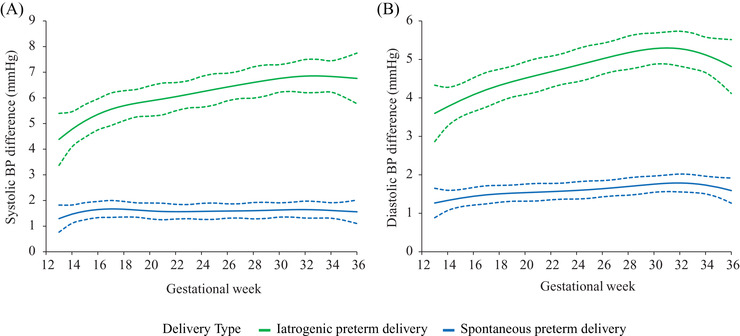
Blood pressure differences against women with term delivery for those with iatrogenic and spontaneous preterm delivery. BP, blood pressure

**FIGURE 3:**
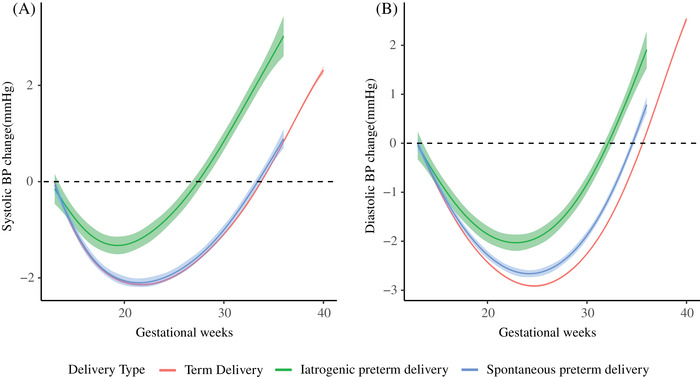
Trajectories of Blood pressure change from 13 weeks of gestation among women with iatrogenic and spontaneous preterm and term delivery. BP, blood pressure

**FIGURE 4:**
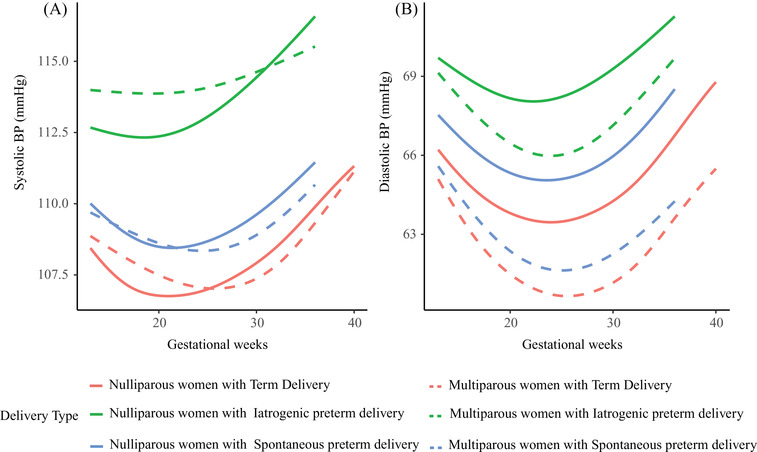
Blood pressure trajectories among nulliparous and multiparous women with iatrogenic and spontaneous preterm and term delivery. All models were adjusted for maternal age, pre‐pregnancy BMI, smoking and passive smoking during pregnancy, parity, maternal income level and education level, infant sex and history of preterm delivery. BP, blood pressure

Linear mixed‐effects models showed that maternal BP trajectories of the three groups were J‐shaped curves. Maternal BP reached the nadir at midgestation and increased consecutively till delivery (Figure 1). Women with iatrogenic preterm delivery had the highest BP from 13 weeks till delivery, followed by those with spontaneous preterm and term delivery (*p *< .001) (Figure 1). Systolic BP for women with iatrogenic preterm reached the nadir at 19 weeks of gestation, while women with spontaneous preterm and term delivery at 22 weeks of gestation, with mean nadir values being 112.72 mmHg (95% CI 112.24∼113.20), 108.36 mmHg (95% CI 108.14∼108.58) and 106.80 mmHg (95% CI 106.74∼106.85), respectively. Diastolic BP for women with iatrogenic preterm, spontaneous preterm and term delivery reached the nadir at 23, 24, and 25 weeks of gestation with mean nadir values being 67.60 mmHg (95% CI 67.19∼68.01), 64.38 mmHg (95% CI 64.19∼64.57) and 62.78 mmHg (95% CI 62.74∼62.82), respectively (Figure 1). The systolic BP difference against term delivery were 4.38 mmHg (95%CI 3.37∼5.40) and 1.29 mmHg (95%CI .77∼1.82) for iatrogenic and spontaneous preterm delivery at 13 weeks of gestation, respectively. These differences gradually increased until delivery. Diastolic BP exhibited a similar pattern (Figure 2). The trajectories of systolic BP change from 13 weeks of gestation were similar for women with spontaneous preterm and term delivery. However, for women with iatrogenic preterm delivery, trajectory of systolic BP change reached the nadir at earlier stage in pregnancy with a smaller change from 13 weeks of gestation. Regarding diastolic BP, women with iatrogenic preterm delivery reached the nadir at earlier stage in pregnancy with a smaller change, followed by those with spontaneous preterm and term delivery (Figure 3). The magnitude of midterm fall from 13 weeks in systolic BP were 1.32 mmHg (95% CI 1.13∼1.51), 2.10 mmHg (95% CI 1.96∼2.23), and 2.14 mmHg (95% CI 2.11∼2.17) for women with iatrogenic preterm, spontaneous preterm and term delivery, respectively. The magnitude of midterm fall from 13 weeks in diastolic BP were 2.03 mmHg (95% CI 1.75∼2.30), 2.66 mmHg (95% CI 2.54∼2.79), and 2.91 mmHg (95% CI 2.88∼2.94) for women with iatrogenic preterm, spontaneous preterm and term delivery, respectively (Figure 3). For nulliparous women, women with iatrogenic preterm delivery tend to have the highest BP trajectory, followed by those with spontaneous preterm delivery and term delivery. For multiparous women, similar patterns were observed (Figure 4).

**FIGURE 5:**
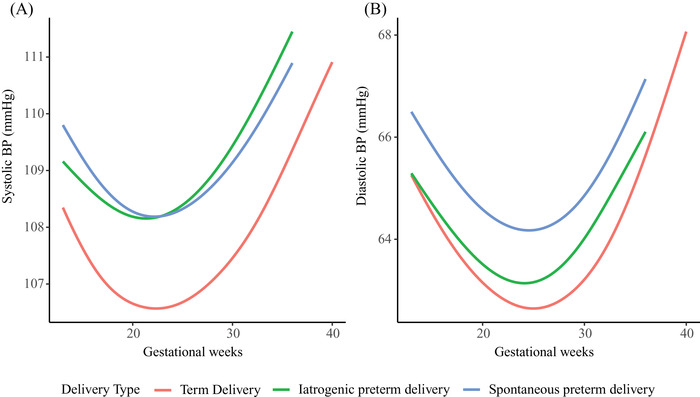
Blood pressure trajectories among women with iatrogenic and spontaneous preterm and term delivery, excluding women with pre‐eclampsia and gestational hypertension. All models were adjusted for maternal age, pre‐pregnancy BMI, smoking and passive smoking during pregnancy, parity, maternal income level and education level, infant sex and history of preterm delivery. BP, blood pressure

Since PE and GH are well‐established risk factors for preterm delivery, we further did the trajectory analysis excluding women with PE and GH. Results in Figure 5 indicated that the trajectory differences between spontaneous preterm delivery and iatrogenic preterm delivery attenuated to null (*p* > .05), while the trajectory differences between preterm delivery and term delivery persisted (*p* < .05).

**FIGURE 6:**
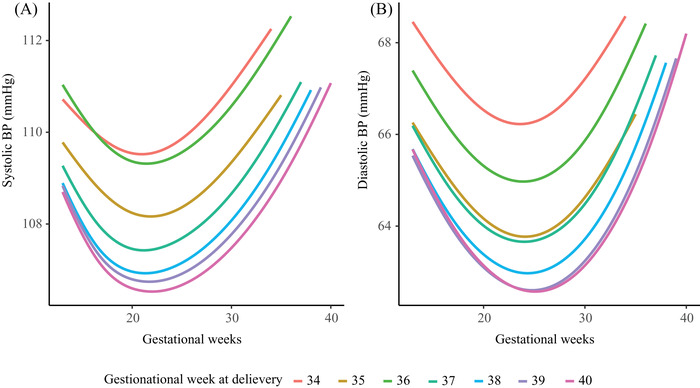
Blood pressure trajectories during pregnancy among women with different gestational length. BP, blood pressure

The BP trajectories analysis for women with different gestational lengths (34, 35, 36, 37, 38, 39, 40 weeks of gestation) showed that women with shorter gestation tend to have higher maternal BP from 13 weeks till delivery (Figure 6).

## 4 DISCUSSION

Women with spontaneous preterm delivery have consistently higher BP from 13 weeks till delivery than those with term delivery. Similar findings were observed for iatrogenic preterm delivery, although women with iatrogenic preterm delivery have higher BP than those with spontaneous preterm delivery. Maternal BP is negatively associated with length of gestation.

To the best of our knowledge, ours is the first to explore the differences in maternal BP trajectory during pregnancy in women with pregnancies ending preterm (spontaneous/ iatrogenic) and at term. Previous studies tended to focus on the narrow spectrum of hypertensive disorders of pregnancy.^23^ Among the few relevant studies, it has been found that both maternal BP at early pregnancy and BP change during pregnancy are related to preterm birth and gestational length, which was consistent with this study.^15^, ^16^ Nevertheless, these studies either did not distinguish between spontaneous and iatrogenic preterm delivery^15^ or only considered spontaneous preterm delivery.^16^ To address this, our study took into account the heterogeneity between subtypes of preterm delivery, adding new evidence that women with spontaneous preterm delivery have consistently higher BP from 13 weeks till delivery than women with term delivery, as those with iatrogenic preterm delivery do. Moreover, the magnitude of the trough for women with iatrogenic preterm delivery is the smallest, followed by those with spontaneous preterm delivery and term delivery, indicating different blood pressure variability (BPV) for different delivery types.

A novel finding of our study is that women with spontaneous preterm delivery experienced consistently higher BP during pregnancy than women with term delivery, while it has been well known that women with iatrogenic preterm delivery are exposed to higher maternal BP during pregnancy. Misra and colleagues^24^ found that both uterine artery resistance index (RI) and umbilical artery RI were significantly higher for spontaneous preterm delivery than term delivery throughout pregnancy, which partially supported our findings.

In this study, women with iatrogenic preterm delivery experienced consistently higher BP from 13 weeks till delivery, followed by those with spontaneous preterm delivery and term delivery. Since BP is a robust marker of subclinical vascular dysfunction among healthy young adults,^25^, ^26^ this might suggest that maternal subclinical vascular dysfunction probably played a role in the occurrence of spontaneous and iatrogenic preterm delivery. Yet the reason why different women end up with different subtypes of preterm delivery remains unclear. Emerging evidence indicates that placenta perfusion insufficiency appears to be the possible cause of preterm delivery.^27^ It is believed that the underlying cause for placenta perfusion insufficiency is suboptimal uterine spiral artery remodeling,^27^ which contributes to improper blood flow to the placenta and thus to pregnancy complications, including preterm delivery.^24^, ^27^ Kim and colleagues^12^, ^13^ indicated that failure of physiologic transformation of spiral arteries was more frequent for preterm delivery than for normal term delivery. Moreover, the suboptimal physiologic transformation of spiral arteries was far more severe in PE. Thus, it is reasonable to postulate that when severe failure of physiologic transformation of uterine spiral arteries is present, suboptimal uteroplacental arteries may predispose pregnancy to reduced placental perfusion and compensatory PE and thus leading to iatrogenic preterm delivery. While the failure of physiologic transformation is less severe, compensatory increased BP may incur spontaneous instead of iatrogenic preterm delivery. Excluding women with PE and GH attenuated the trajectories differences between iatrogenic preterm delivery and spontaneous preterm delivery to null. This might partially support the abovementioned vascular mechanism, which warrants further thorough examination of the placenta in future studies.

Despite substantial research directed at understanding the pathophysiology of preterm delivery and evaluating strategies for prevention, prevalence has continued to rise from 5.36% in 1990–1994–7.04% in 2015–2016.^28^ Previous attempts at risk prediction of preterm delivery showed limited predictive capacity.^29^ Most of these studies focused on risk factors during the second half of pregnancy,^30^ which might limit the potential for early intervention. Moreover, risk factors such as cervical length and fetal fibronectin have been proved to be of high predictive utility only in high‐risk population.^30^ To make things worse, the majority of women who deliver prematurely have no known risk factors and over 50% of preterm delivery occurred in low‐risk population.^31^ As we can see in this study, maternal BP trajectories differ for spontaneous preterm delivery, iatrogenic preterm delivery and term delivery. Other than different initial BP, the magnitude and velocity of the trough at midpregnancy also varies, resulting different BPV. However, numerous studies did not incorporate BPV as a predictor for preterm delivery.^29^, ^32^, ^33^, ^34^, ^35^ BP is an easily measured and routinely collected information during pregnancy, future studies should examine the possibility of BPV as a risk factor for preterm delivery. This would be beneficial to risk classification and thus identify more at‐risk population, especially for those pregnant women with no known risk factors.

A strength of our study is the relatively large sample size. A large sample size provides sufficient statistical power to explore the association between maternal BP during pregnancy and gestational length. Also, the large sample size and the prospective design secure high precision of our findings. Last but not the least, multiple measurements of BP per women throughout pregnancy permits robust and accurate trajectory analysis. However, our study has some limitations that are worth noticing. Firstly, the participants in the Born in Guangzhou Cohort Study are all Chinese,^18^ which may limit the generalization of our findings to other populations. Secondly, given the fact that the majority of preterm delivery cases in the present study were between 34 and 36 completed gestational weeks, the exploration of maternal BP trajectory with the severity of preterm delivery was limited. Nevertheless, the dose‐response relationship of BP throughout pregnancy with gestational length indicates a plausible robust association between maternal BP and preterm severity. Thirdly, clinical causes for spontaneous and iatrogenic preterm delivery are numerous. Given relatively low cases in each category, out study did not bear enough statistical power to subgroup analysis. Fourthly, the unavailability of pre‐pregnancy BP and tremendous scarcity of BP prior to 13 weeks of gestation made it impossible to explore the BP change at earlier stage. Fifthly, antihypertensive medication during pregnancy was not corrected in this study. In clinical practice, clinicians are cautious about initiation of antihypertensive medication during pregnancy in consideration of fetal growth restriction^36^ unless it's severe hypertension. Therefore, although medication during pregnancy is not adjusted for, the observed differences among three different groups might be an underestimation of the actual differences. Sixthly, although the BP among three groups were significantly different, the causality between BP and preterm delivery still remained to be elucidated. Lastly, previous study has shown a seasonal pattern in preterm delivery. However, the change in the rate of preterm delivery in different season was quite minimal,^37^ which was unlikely to change the observed association in this study. Nevertheless, we admit that there were still possibilities of residual confounding.

## 5 CONCLUSION

In summary, women with spontaneous preterm delivery experience consistently higher BP during pregnancy than women with term delivery, which also applies to iatrogenic preterm delivery. Further, higher maternal BP during pregnancy is associated with shorter gestational length.

## CONFLICT OF INTEREST DISCLOSURE

The authors declare that they have no conflict of interests.

## PATIENT CONSENT STATEMENT

Written informed consent was obtained from all participants before participating in Born in Guangzhou Cohort Study.

## AUTHORS' CONTRIBUTIONS

Xiu Qiu, Huimin Xia, Jianrong He and Fanfan Chan contributed to conception, design and development of methodology. Fanfan Chan, Songying Shen, Peiyuan Huang, Xueling Wei, Jinhua Lu, Lifang Zhan and Xiaoyan Xia contributed to data collection. Fanfan Chan, Xiu Qiu, Jianrong He, Kar Keung Cheng, Shakila Thangaratinam and Ben Willem Mol contributed to analysis and interpretation of the data. All authors approved the manuscript and agree to be accountable for all aspects of the work.

## Supporting information

Supplementary Figure 1. Flowchart for the selection process of the participants in the present study.
