# Supplementary material for: Blood pressure trajectories during pregnancy and preterm delivery: A prospective cohort study in China
Source: J Clin Hypertens (Greenwich). 2022 Jun 1;24(6):770–8. doi: 10.1111/jch.14494 (PMC9180333; doi:10.1111/jch.14494)
Supplement: Supplementary file 1 — Supplementary Figure 1. Flowchart for the selection process of the participants in the present study. [file JCH-24-770-s001.pdf]

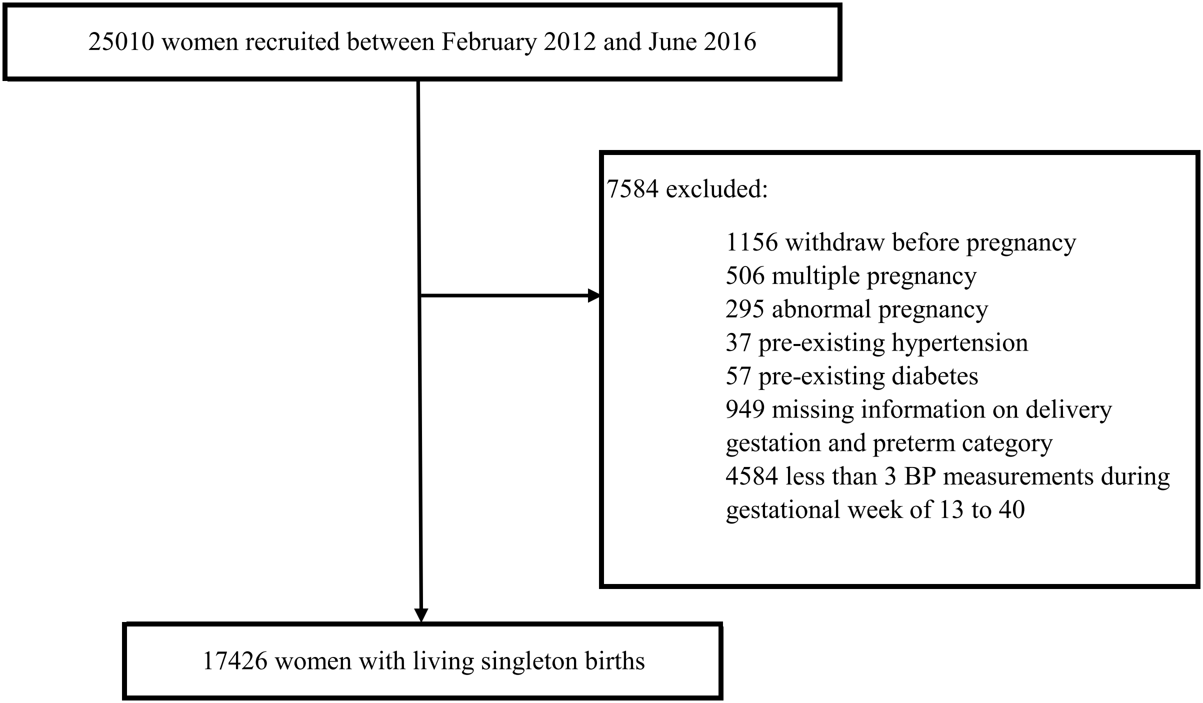
25010 women recruited between February 2012 and June 2016
7584 excluded:
1156 withdraw before pregnancy
506 multiple pregnancy
295 abnormal pregnancy
37 pre-existing hypertension
57 pre-existing diabetes
949 missing information on delivery gestation and preterm category
4584 less than 3 BP measurements during gestational week of 13 to 40
17426 women with living singleton births
